# What is treatment free remission in chronic myeloid leukemia?

**DOI:** 10.18632/oncotarget.23398

**Published:** 2017-12-18

**Authors:** Delphine Rea, Gabriel Etienne, François-Xavier Mahon

**Affiliations:** Institut Bergonie Cancer Center, Department of Hematology, Universite Bordeaux, Bordeaux, Aquitaine, France

**Keywords:** chronic myelogenous leukemia, tyrosine kinase inhibitors

Chronic myeloid leukemia (CML) has become a model for molecular targeted therapy [1]. It is characterized by a cytogenetic marker, the Philadelphia (Ph) chromosome and its molecular counterpart the *BCR-ABL1* fusion gene. This leukemia-specific molecular marker encodes a protein tyrosine kinase, BCR-ABL1, which is considered as the driver of the leukemic process. This discovery led to the development of BCR-ABL1 tyrosine kinase inhibitors (TKIs), which definitely resulted in a dramatic improvement of CML patient’s outcome. The life expectancy of TKI-treated CML patients has recently been reported to be close to that of the general population [2]. Under TKI treatment, a substantial part of the patients with CML in chronic phase (CP) achieve a so-called deep molecular response (DMR), which is identified by measuring blood *BCR-ABL1* transcripts levels with real-time quantitative polymerase chain reaction (RQ-PCR).

Until now the recommendation for CML patients with TKI therapy was to continue the treatment indefinitely. However, there are plenty of good reasons for trying to stop TKI. The off-target effects of TKI and severe adverse drug reactions have been largely reported. Notwithstanding the fact that these side effects impair quality of life, some of them potentially might modify also overall and healthy life expectancy through TKI-related events like pulmonary arterial hypertension, pleural effusion or cardiovascular events [3]. In addition, use of TKI is forbidden in pregnant women due to malformative effects and children growth is also altered by TKI in pediatric CML cases. Patients’ requests are also important and the question of whether TKI is a lifelong treatment is frequently asked.

Many clinical trials have demonstrated the feasibility of stopping TKI in patients with durable DMR beyond MMR [4–5]. The convincing results of all of these studies have validated the concept of treatment free remission (TFR) which is becoming the main criteria of some clinical trials in CML. The *sine qua non* condition for proposing to stop TKI is the achievement a sustained DMR. A certified laboratory is necessary to perform and validate robust molecular monitoring for safety of TFR studies. By the way, the results of the many independent TFR studies performed to date are highly reproducible [6]. About half of the patients who are eligible for TKI discontinuation remain treatment-free while the others recovered optimal responses upon therapy re-introduction. Most of molecular recurrences occur within the first 6 months after TKI cessation. Stopping treatment is safe since patients who need to be re-treated with TKI after a molecular recurrence are sensitive again to the treatment with a new induction of a DMR. Unexpectedly, a peculiar transitory and benign TKI withdrawal syndrome has been reported but the mechanism is unknown [7]. The key question resulting from TFR trials is the evidence of persisting leukemic cells even in patients who don’t exhibit a true relapse regardless of its definition.

The number of patients who are stopping TKI treatment is increasing over time and it may even be even possible to stop treatment several times within a single patient. This has been proven by a multicenter study entitled RE-STIM, in which a 2^nd^ TKI discontinuation attempt was proposed after failure of a 1st attempt. Eligible patients, i.e. those with a sustained DMR, remained in TFR after a 2nd discontinuation attempt in about one third of cases [8].

Although very long-term TFR experience is limited, substantial knowledge accumulated during the last years justifies moving TFR strategies from research to clinical practice.
